# Bioengineered Enzymes and Precision Fermentation in the Food Industry

**DOI:** 10.3390/ijms241210156

**Published:** 2023-06-15

**Authors:** Fatma Boukid, Seedhabadee Ganeshan, Yingxin Wang, Mehmet Çağlar Tülbek, Michael T. Nickerson

**Affiliations:** 1 ClonBio Group Ltd., 6 Fitzwilliam Pl, D02 XE61 Dublin, Ireland; fboukid@clonbioeng.com; 2 Saskatchewan Food Industry Development Centre, Saskatoon, SK S7M 5V1, Canada; lwang@foodcentre.sk.ca (Y.W.); mtulbek@foodcentre.sk.ca (M.Ç.T.); 3 Department of Food and Bioproduct Sciences, University of Saskatchewan, Saskatoon, SK S7N 5A8, Canada; mtn620@mail.usask.ca

**Keywords:** enzyme engineering, synthetic biology, precision fermentation, gene editing, food industry, plant proteins

## Abstract

Enzymes have been used in the food processing industry for many years. However, the use of native enzymes is not conducive to high activity, efficiency, range of substrates, and adaptability to harsh food processing conditions. The advent of enzyme engineering approaches such as rational design, directed evolution, and semi-rational design provided much-needed impetus for tailor-made enzymes with improved or novel catalytic properties. Production of designer enzymes became further refined with the emergence of synthetic biology and gene editing techniques and a plethora of other tools such as artificial intelligence, and computational and bioinformatics analyses which have paved the way for what is referred to as precision fermentation for the production of these designer enzymes more efficiently. With all the technologies available, the bottleneck is now in the scale-up production of these enzymes. There is generally a lack of accessibility thereof of large-scale capabilities and know-how. This review is aimed at highlighting these various enzyme-engineering strategies and the associated scale-up challenges, including safety concerns surrounding genetically modified microorganisms and the use of cell-free systems to circumvent this issue. The use of solid-state fermentation (SSF) is also addressed as a potentially low-cost production system, amenable to customization and employing inexpensive feedstocks as substrate.

## 1. Introduction

In recent years, advances in synthetic biology have led to the rapid synthesis of bioengineered enzymes with applications in the food industry. Prior to modern molecular tools for enzyme engineering, classical enzyme modification strategies included mutagenesis and directed evolution, and extensive laborious screening to identify desired enzyme activity and functionality. While such approaches are still relevant and used, they have been largely superseded by high throughput gene editing technologies and combinations of synthetic biology for using microbial cells as microbial cell factories for more sustainable healthy food production. The bioengineering of food enzymes via fermentative processes has received particular attention, as it is considered a clean-label processing aid by the food industry.

**Figure 1:**
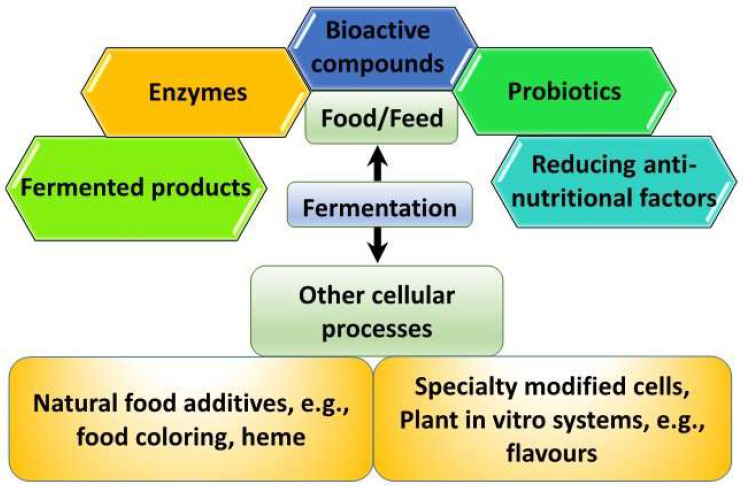
Fermentation-enabled food valorization potential.

Fermentation entails metabolic processes by which bacteria, yeast, fungi, or algae use carbohydrate sources to effect changes in substrates to be used for consumption, food additives, or become the source of food or supplements themselves (Figure 1). Many of the changes occurring during fermentation contribute to alterations in flavor, texture, and shelf-life extension, besides health-beneficial effects. However, fermentation technology, for the rapid production of designer molecules ensuing from synthetic biology has gained prominence in recent years. Touted as one of the emerging technology breakthroughs of the fourth industrial revolution, synthetic biology including other associated tools involving AI, bioinformatics, systems biology, and computational biology have paved the way for what is now known as precision fermentation [1]. It is essentially perceived as an advanced fermentation system due to the precise production of specific molecules under very controlled manufacturing processes to maximize the yield of desired products and minimize cost [2]. Unlike traditional fermentation, which includes products such as kimchi, yogurt, and tempeh, precision fermentation relies on more intensive control and manufacturing processes and investment in more specialized technology. Biomass fermentation, on the other hand, involves the cultivation of microorganisms capable of high-yield biomass with high nutritional contents, if targeted for food ingredient usage.

Considering the importance of enzymes in the food industry and the significant roles synthetic biology and precision fermentation are and will be playing in this sector in the foreseeable future, the objective of this review is to highlight the state of bioengineered enzymes in the food industry. Despite its impact on the food industry, enzyme-engineering technology is not without its challenges, and it is important to put those into perspective, especially with regard to safety and scale-up production. With the debate around the use of genetically modified microorganisms (GMO) still ongoing, we discuss gene editing which is likely to assuage some of those safety concerns. More importantly, we put into perspective the use of cell-free systems (CFS) for engineered enzyme production to circumvent the perceived GMO-associated risks. Due to the high costs associated with the manufacture of engineered enzymes by submerged liquid fermentation, be it conventional or precision, we also suggest a rethink of the current strategy of precision fermentation and start focusing on a solid-state precision fermentation (SSPF) strategy, since solid-state fermentation is low-cost, amenable to customization and easily adaptable to the use of inexpensive feedstocks.

## 2. Strategies and Challenges for the Development of Engineered Enzymes in the Food Industry

**Figure 2:**
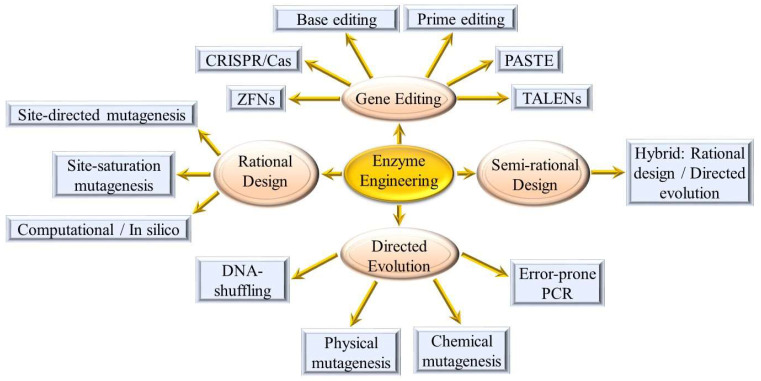
Enzyme engineering strategies.

The ubiquitous nature of enzymes and their important roles in the alteration of food components cannot be overemphasized due to their multifunctional properties for the improvement of product quality and stability [3]. The major bottleneck in the industrial use of naturally occurring enzymes is generally associated with their low adaptability and stability under harsh food processing conditions (e.g., high/low pH and temperatures) [4,5]. To overcome these limitations, enzyme engineering strategies (Figure 2) have been developed to design new enzymes with improved stability, specificity, and activity while increasing biocatalysis and reducing energy consumption [6,7]. 

Directed evolution and rational design are the two well-established engineering approaches to designing new enzymes with tailor-made biocatalytic properties [8,9]. The selection of the appropriate approach relies on the level of knowledge and understanding of the target enzyme structure and function [10]. The selected best variant is then generally expressed in bacteria (e.g., *Escherichia coli*, *Bacillus*, and lactic acid bacteria), filamentous fungi (e.g., *Aspergillus*), and yeasts (e.g., *Pichia pastoris*) and then screened for functionality [11,12]. Engineered enzymes such as amylases, xylanases, lipases, proteases, cellulases, and pectinases are utilized in a wide range of food applications such as bakery, dairy, brewery, and confectionary (Table 1). With the advent of advanced gene editing methodologies, further opportunities for enzyme engineering were created. To put these different approaches into perspective, a brief overview is provided in the subsequent sections. 

### 2.1. Directed Evolution

Directed evolution relies on creating mutant libraries via iterative random mutagenesis (using PCR techniques, chemical mutagenesis, UV irradiation, or DNA-shuffling techniques) [12,34,35]. Systematic screening and evaluation processes are required to identify the enzymes having the desired properties [36]. For this approach, knowledge about enzyme structure is not necessary. However, the experimental burden of making and screening a high number of random mutants is highly resource and time intensive [37]. Advances in de novo computational design such as machine learning have accelerated directed evolution by screening in silico combinatorial libraries of mutations to predict the function from the sequence and thus leading to the generation of new enzymes with optimized functions [38,39,40]. 

For instance, directed evolution coupled with a high-throughput robotic screen was employed to overcome the limitations of naturally obtained amylases such as low pH and high temperature. Using error-prone PCR, novel α-amylase (Novamyl) from *Bacillus* sp. (TS-25) and *Bacillus licheniformis* showed increased thermostability and acid pH tolerance [8]. When added to bread dough, these engineered enzymes improved fresh bread quality and delayed staling (by reducing hardness, improving elasticity, and maintaining organoleptic features) [12]. Site-directed evolution also improved the thermostability and acid resistance of α-amylase from *Rhizopus oryzae* resulting in increased starch hydrolysis (1.61-fold) [9]. In silico approach resulted in α-amylase from *Bacillus cereus* GL96 with high thermostability (70 °C) and stability over a range of pH from 4 to 11 [13]. *Bacillus amyloliquefaciens* xylanase (reBaxA50) made by error-prone touchdown PCR increased the catalytic efficiency and the stability under thermal and extreme pH treatment compared to the wild-type [14]. Tailored xylo-oligosaccharides having prebiotic benefits were obtained through the hydrolysis of xylan-rich sources using engineered xylanase [15]. Using classical error-prone PCR, engineered *Pseudomonas fluorescens* and *Penicillium cyclopium* lipase were produced with enhanced thermostability and alkali stability which expanded their uses as catalysts in bakery and dairy [16,17]. A new β-galactosidase obtained using error-prone PCR had improved specific activity for lactose hydrolysis in milk processing [18]. Liu et al. [19] developed *Bacillus alcalophilus* alkaline protease using error-prone PCR with high cold adaptation suitable for cold-temperature food processing. A new transglutaminase enzyme from *Streptomyces mobaraensis* using site-directed mutagenesis also showed improved thermostability and specific activity for use in different applications such as bakery [20]. 

### 2.2. Rational Design

Rational design requires in-depth knowledge of the target enzyme properties (structure, catalytic mechanism, active site, and their distribution to function) [41]. To produce enzymes with desired properties, specific DNA regions encoding specific amino acids (identified using structural analysis and computational modeling) related to the desired activity/functionality are replaced, inserted, or deleted [42]. Site-directed mutagenesis is the most commonly used rational design approach, and it is based on the substitution of a specific amino acid to design enzymes with improved functionality [43,44]. For instance, a serine peptidase from *Pseudomonas aeruginosa* resulted in improved thermal stability and catalytic efficiency compared to the wild type [21]. Xylanase generated using site-directed mutagenesis enhanced substrate specificity owing to the production of tailor-made xylo-oligosaccharides [22]. In bread making, the use of engineered xylanase improved the performance of wheat flour using a lower dosage compared to the wild-type and resulted in increased bread volumes [45]. Recombinant β-glucanase from *Bacillus* showed enhanced catalytic efficiency, halostability, and thermostability [23,24]. Modified *Candida rugosa* lipase isozymes LIP1 showed high catalytic efficiency to produce fatty acid esters and diglycerides, which can be used as food emulsifiers [25].

Site-saturation mutagenesis is another rational method based on the substitution of a specific amino acid with the other 19 possible amino acids. De novo approaches are gaining increased interest due to their ability to create novel enzymes displaying diverse functions that do not occur naturally [46,47]. Computer-aided enzyme simulation enabled to redesign of Cel9A-68 cellulase from *Thermobifida fusca* with improved cellulase activity. Such features could be extremely relevant for cellulose degradation in the brewery sector [26]. A lipase from *P. aeruginosa* PAO1 was recently computationally “reverse-engineered” using proline mutations. This mutation enabled the creation of new variants with increased activity and stability to be used for flavor development in dairy products [27,48]. Several studies focused on upgrading xylanase activity due to its relevance in bread making. GH11 xylanase from *Neocallimastix patriciarum* had improved thermostability and kinetic efficiency [28]. In a recent study, GH11 xylanase from *Bacillus* sp. strain (T82A) was selected out of 576 strains as the most thermostable variant [29]. GH11 mutated sites from *Aspergillus niger* had the highest catalytic activity and thermostability due to improved binding affinity of enzyme and substrate [30]. Artificial intelligence tools such as protein “Hallucination”, ProteinMPNN, DenseCPD, and Unsupervised Learning Methods (ULM) could further boost the implementation of de novo features in tailored enzymes to fit conventional and emerging food applications [49,50,51].

### 2.3. Semi-Rational Design

Semi-rational design is a hybrid approach combining the benefits of rational design and directed evolution [52,53]. This approach uses existing libraries (like rational design) to select a specific promising sequence, but the specific amino acid related to the targeted function is unknown [54,55]. The mutation of single amino acids or a combination of them generates new mutants to be screened and evaluated like in directed evolution to identify the desirable variant in the library [54]. Smart libraries created by semi-rational design contributed to the improvement of the catalytic activity of type II ASNase from *Bacillus licheniformis* [31]. Type II ASNase can be used to mitigate acrylamide in fried potato products, bakery products, and coffee without affecting the appearance, quality, and taste of the product [56]. However, the low thermal stability of the wild type limits its use. Recently, structural alignment and molecular dynamic simulation demonstrated that new mutants made using semi-rational design had high catalytic efficiency, structural stability, and strong substrate binding [31]. Semi-rational mutagenesis and low-throughput screening also resulted in a double mutant (L341I_Q345S) of sucrose phosphorylase with a selectivity of 95% for kojibiose [32]. Kojibiose has several health benefits including delayed glucose release and a boost in SCFA production. In addition, it can be used as a sweetener in confectionery products [57]. Using a semi-rational design, a new mutant of cellobiose 2-epimerase with improved thermostability and catalytic efficiency was used in the production of lactose-based prebiotics [33].

### 2.4. Gene Editing

Molecular engineering of enzymes for improved functionality and activity is certainly a more tailored approach compared to screening for mutant cell lines or by directed evolution. However, the advent of more precise genome editing tools (Figure 2) has enabled faster generation of altered gene functions. The conventional restriction enzyme-based recombinant technology has largely been superseded by newer genome-editing tools such as zinc finger nucleases (ZFNs) [58], transcription activator-like effector nucleases (TALENs) [59], Clustered regularly interspaced short palindromic repeats (CRISPR)/CRISPR-associated (Cas9) systems [60], base editing [61], prime editing [62] and Programmable Addition via Site-specific Targeting Elements (PASTE) [63]. With such a repertoire of techniques, the choice of approach to use is dependent on the resources and expertise available. In recent years, a number of gene editing service providers have also emerged, thus allowing for faster results, and circumventing the need for less well-equipped laboratories to invest extensively in establishing such technologies.

While initially much of the efforts were directed towards achieving high levels of gene expression through multiple integrations [64], strong promoters, or inducible promoters [65], the focus has shifted to the gene editing techniques mentioned above to improve functionality and activity. For example, to disrupt proteases and enhance the activities of heterologous expression of pullulanase in *Bacillus subtilis* strain WS5, CRISPR/Cas9 was used [66]. CRISPR/Cas9 has also been used in fungi due to their high efficiency for enzyme production. To prevent Non-Homologous End Joining (NHEJ) DNA in *Penicillium subrubescens* and generate a strain capable only of homologous recombination when used as a parental strain for site-specific recombination, CRISPR/Cas9 was used to inactivate the *Ku70* gene responsible for NHEJ repair system [67]. CRISPR has also been used to engineer microbes for the production of high-value products such as squalene, which is illegally extracted from shark liver or recovered at low yields from plants. Park et al. [68] were able to engineer *Corynebacterium glutamicum* using CRISPR interference to produce squalene from glucose by altering multiple key enzymes in the methylerythritol 4-phosphate (MEP) pathway. Heterologously expressed genes, followed by CRISPR/Cas9 editing have also been done to increase enzyme activity. For example, the trehalase enzyme, which catalyzes the conversion of one trehalose molecule to two glucose molecules, was transferred to *Aspergillus niger* from *Myceliophthora sepedonium* by homologous recombination and exhibited trehalase activity of 406.44 U/mL [69]. However, upon adopting a multi-copy knock-in expression strategy using CRISPR/Cas9 editing, trehalase expression in *Aspergillus niger* increased about five-fold compared to the homologously recombined wild-type *trehalase* gene.

The gene editing technologies are certainly promising in advancing metabolic circuitry alterations for flavor, texture, nutritional, and health beneficial value enhancement. The recent market release of the CRISPR-edited gamma-aminobutyric acid (GABA)-enriched tomato is the first such product in Japan [70]. GABA is promoted as a health supplement and was produced due to the increased activity of the enzyme glutamic acid decarboxylase to convert glutamate to GABA upon suppression of the calmodulin-binding domain gene. Prevention of food losses due to enzymatic browning has also been investigated using gene editing. Enzymatic browning is due to the enzyme polyphenol oxidase (PPO), which catalyzes the oxidation of phenolic compounds to dark-colored quinones. In potato tubers, edited PPO genes led to a 69% reduction in PPO activity and a 73% reduction in enzymatic browning [71]. While these gene-edited crop plants provide powerful new approaches for targeted enzyme modification for in situ expression, the acceptance of these gene-edited crops are still subject to debate. In the meantime, to circumvent these potential roadblocks, engineering enzymes using synthetic biology and gene editing is generally regarded as safe (GRAS) microorganisms for the food industry remains the best strategy.

### 2.5. Safety Challenges

One of the main safety aspects of developing engineered enzymes relies on ensuring that the used microorganism must qualify as a Qualified Presumption of Safety (QPS) organism and be able to produce a specific food enzyme at a constant level [72]. Even the use of GRAS microorganisms is not without its perceived safety risk over contamination and allergenicity potential. Thus, risk assessment of the safety of the genetic modification includes the evaluation of the source of the enzyme, the introduced sequences, the characteristics of the parental and recipient microorganisms, and the genetic modification process (e.g., the absence of antibiotic resistance genes). 

For enzyme engineering, the alteration of amino acid sequences does not typically induce variability in the catalytic site or the identity of the enzyme. Nevertheless, the change of amino acid sequences may increase their allergenic potential. It was reported that genetically engineered enzymes could elicit immediate-type sensitization [73,74]. This urges regular surveillance of commercial enzymes through the development of specific IgE assays. For enzyme manufacturing, protein-engineered enzymes are produced using the same processes as the wild-type, and thus have similar process risks as the wild-type [75]. For the host microorganism, there is a list of strains such as *Bacillus subtilis*, *Bacillus amyloliquefaciens*, *Bacillus licheniformis*, *Aspergillus niger*, and *Aspergillus oryzae* with a long history of safe food uses and published in reports and opinions prepared by international organizations such as FAO/WHO, FDA and the European Food Safety Authority (EFSA) [76]. Safety measures and risk assessment procedures are continuously updated by established organizations to ensure the safety of newly engineered enzymes. Genome-editing tools such as CRISPR/Cas9 can precisely and safely target specific changes in microorganisms without introducing exogenous genetic elements [77]. Although the use of CRISPR is legal in the USA, the EU still applies the GMO Directive to the genome-edited organisms. Recently, EFSA concluded in their opinion that the risk assessment methodology and the existing guidelines in the GMO legislation are sufficient to prove the safety of genome-edited organisms [78]. However, the current GMO regulation was developed for organisms produced using established methods of genetic modification and not gene editing. Thus, suitable documentation and guidelines are still needed in the EU to regulate this sector [79].

### 2.6. Cell-Free Systems to Circumvent GMO Concerns

With the abundance of targets for modification and more recently, the availability of artificial intelligence-based target identification and selection for modification, bioinformatics, systems biology, and computational biology tools [2], there is no limit to the expanse of the gene-editing technology for improving food quality. Irrespective of the debates surrounding GMO safety risk assessments and evolving regulatory frameworks, gene editing for enzyme functionality improvement will continue to be significant in the foreseeable future. In fact, it is likely that cell-free systems will become an alternative to GMOs, thus eliminating the risks and controversies surrounding such microorganisms [80,81]. With the fourth wave of biocatalysis heralded by the advent of advances in molecular biology, synthetic biology, sequencing technologies, gene editing, bioinformatics, and computational biology [82,83], it was the logical next step for considering cell-free systems (CFS) for engineered enzyme production. CFS, is in itself not new, as it has been reported over six decades ago [84], and the availability of a repertoire of platform technologies has provided renewed interest in its potential applications [85]. Although still at an early stage, progress is being made to subsequently develop scalable production and manufacturing platforms. CFS essentially involves the production of a protein in a matrix of cell lysates, crude extracts, or synthetic cocktails in the presence of the transcriptional and translational components outside of an intact cell [86]. Mimicking the biological process outside of the cell offers advantages such as more control and monitoring of reactions, efficient protein folding, and post-translational modifications [87]. Furthermore, as mentioned above, safety risks associated with GMOs are eliminated.

While it is unlikely that CFS will supplant current food enzyme production approaches through conventional or precision fermentation, CFS may offer advantages in certain specific situations in the production of secondary metabolites, proteins, or even vitamins. For example, myo-inositol (Vitamin B8) was produced using a four-enzyme pathway system supplemented with two other enzymes for the conversion of starch into myo-inositol [88]. This non-fermentative production of myo-inositol was successfully demonstrated at a 20,000 L scale, although the enzymes themselves were produced via fermentation [88]. Of note in this study was also the demonstration of myo-inositol production using thermophilic and hyperthermophilic enzymes, which enabled the reactions to be conducted at high (70 °C) temperatures, thus preventing any stability or reaction kinetics hindrance. Using a similar strategy with hyperthermophilic enzymes, sucrose [89] and D-xylose [90] were used as substrates instead of starch for the production of myo-inositol. More recently, pigmented metabolites such as lycopene, indigoidine, betanin, and betaxanthins, which have applications in the food, cosmetic, textile, and pharmaceutical industries, were synthesized by co-expression of three enzymes in the presence of tobacco BY-2 cell suspension lysates [91]. The fact that these are visually discernible metabolites enables monitoring their accumulation during synthesis. The scale-up production of these metabolites is yet to be demonstrated. However, the production of cell lysates from the tobacco BY-2 cell suspension cultures can adequately be performed in large-scale bioreactors just as the large-scale quantities of the enzymes needed to catalyze the reaction.

## 3. Engineered Enzymes for Improved Plant-Based Beverages and Meat Alternatives

When discussing engineered enzymes in the food industry, it is important to consider their applications in the plant-based beverage and meat alternative context as well. Recent trends indicate an exponential growth in the use of plant proteins. This shift in preference by consumers for more plant-based diets stems from health, sustainability, and/or ethical considerations. However, several plant proteins have nutritional (lacking essential amino acids and low digestibility), functional (e.g., low solubility), and organoleptic (off-flavor and bitter taste) limitations [92]. Thus, there is a need to design enzymes to address the limitations of plant-based ingredients in the food industry due to their increasing demand. The development of new enzymes for debittering can be through engineering new proteases able to cleave plant proteins during extraction at specific sites, thus, limiting the release of low molecular weight peptides containing hydrophobic amino acid. Engineered enzymes could also serve to generate specific plant-based peptides with antioxidant, immunomodulatory, antihypertensive, and antimicrobial activities [93]. The yield and the purity of plant proteins are also closely related to the enzymes used during the extraction. In starch biorefinery, where engineered enzymes such as xylanase, amylase, and pectinase are used to hydrolyze starch, most of the proteins are released as a result of this process [94,95]. Another critical point is plant protein functionality. Engineered enzymes could serve as post-processing aids to improve the solubility of plant proteins and therefore their functionality. Custom-made modifications could expand the uses of plant proteins and further boost their markets beyond soy and pea proteins. For instance, proteins with allergenic and toxic propensities such as in faba and lupin could be reduced through engineered enzymes capable of degrading such proteins to obtain safer and more functional proteins able to compete in the protein market. 

The development of engineered enzymes to produce high-quality plant-based beverages is also gaining interest. Engineered α-amylase can catalyze the hydrolysis of starch to obtain simpler and more digestible sugars, thus creating a natural sweetness. These enzymes could modulate the viscosity to obtain plant-based milk with improved texture. Engineered proteases could contribute to hydrolyzing proteins to increase their bioavailability and thus their digestibility. This will boost the nutritional quality of milk alternatives having lower nutritional quality compared to animal-based products. In cheese making, 80% of the currently used rennet is genetically engineered chymosin [96]. These recombinant milk-clotting enzymes have also served in creating plant-based cheeses. For instance, recombinant cardosin can produce large quantities of proteases to be used in making vegan cheese [97]. Precision fermentation has enabled the production of protein, enzymes, fats, and vitamins similar to those present in milk, cheese, and ice cream. Food technology startups such as Formo and Real Deal Milk, are using this technology to deliver equivalent taste, texture, and nutrition as conventional dairy products. Vitamins and minerals lacking in plant sources are being produced using precision fermentation and used for fortifying plant-based beverages to upgrade their nutritional quality. 

In meat alternatives, engineered transglutaminases could contribute to improving the texture through the cross-linking of lysine between the γ-glutamyl residues and the ε-amino groups of lysine residues to extend protein chains [98]. Transglutaminase was added in textured protein formulations and showed a significant impact on textural properties such as elasticity and chewiness depending on the dose and the type of proteins [99]. Engineered transglutaminase can modulate the organoleptic properties of textured proteins (low moisture and high moisture). Engineered transglutaminase with specific crosslinking sites could drastically improve the bite and the mouthfeel of the meat alternatives. Engineered proteases could also play a role in improving the taste and contribute to creating an umami flavor such as through the application of the protease, Novozymes Protana^®^. Amano Enzyme developed a natural protease, Umamizyme™ Pulse, to hydrolyze plant proteins to produce a meaty (umami) flavor in proteins by increasing the glutamic acid and reducing the bitterness. Through protein engineering, the production of such enzymes could be optimized in terms of yield, cost, and sustainability. Established enzyme manufacturers can lead this sector by using their expertise to develop a custom portfolio of enzymes. Advanced computational biology and protein engineering would contribute to the design of new variants with novel functionalities [100]. Precision fermentation enabled the production of “heme” proteins such as soy leghemoglobin using *Pichia pastoris* and Hemami™ to imitate the flavor and color of meat.

## 4. Fermentation Scale-Up Challenges

Despite the advances in enzyme engineering, synthetic biology, gene editing, and recently cell-free systems, the limitation to scale-up production and manufacturing remains a significant challenge. Scale-up challenges in fermentation, be it submerged or solid-state, are not new. The technology has been in existence for a long time, but the knowledge base or expertise has been lacking. From a technical perspective, scale-up challenges include process development and optimization by judiciously selecting appropriate growth media, substrates, feedstocks, incubation temperature, and pH, and also considering the complexity of the fermentation process and downstream processing to obtain purified products [101,102]. Although precision fermentation allows for the specific molecule of interest to be produced efficiently, purification strategies can sometimes be still very complex. For many start-ups, feasibility and sustainability studies may not have been conducted a priori, and at the scale-up stage production costs become prohibitive [103]. Furthermore, scale-up facilities such as contract research organizations (CRO) or contract manufacturing organizations (CMO) are still not widely available or accessible.

To address scale-up challenges in fermentation, Ganeshan et al. [101] recently proposed adopting a Process Hazards Analysis (PHA) approach. PHA is essentially taking an approach to fermentation production by applying manufacturing practices widely employed in industrial engineering manufacturing by systematically, comprehensively, and analytically reviewing a process to enable the identification of process and operational hazards and their impacts [104]. In the food industry risk mitigating strategies such as Good Manufacturing Practices (GMP), Good Hygiene Practices (GHP), Quality Management Systems (QMS), and Hazard Analysis and Critical Control Points (HACCP) are already being followed and have been proven to be robust. However, in terms of fermentation, a more holistic approach needs to be followed to mitigate risks at every step of the process.

### 4.1. Submerged Liquid Fermentation (SmF)

Prior to embarking on scale-up production, it is imperative that a thorough investigation of available resources needs to be conducted. It is often suggested that submerged fermentation (SmF) scale-up be conducted in two stages: a pilot scale from 100–10,000 L and a demonstration scale from 10,000–100,000 L [105]. While this is true, there is always a tendency to expedite processes to achieve scale-up without adequate process development and optimization at smaller scales in the range of 10–50 L, and importantly inadequate technology transfer package development [101]. Consequently, sub-optimal yields are encountered, which contribute to uneconomical and unsustainable production [101]. To some extent, the yields can be addressed due to SmF being adaptable to continuous operation compared to a batch operation. This continuous fermentation process for enzyme production, besides allowing for increased yield, can also lead to cost savings. However, there is a risk of contamination as it involves replenishing the nutrient medium and harvesting at regular intervals.

While initial considerations of the aspects mentioned above are critical, it is important to note that SmF uses significantly large volumes of liquids and to this end, associated downstream processes to concentrate products need to be considered as well. For enzyme scale-up production, this implies an additional cost for purification and concentration, although SmF is preferred because of generally higher yields and lower risks of contamination [106]). With the advent of precision fermentation, yields of enzymes and risks of contamination are not of major concern, although concentration and purification of enzymes from the nutrient broth still need to be addressed.

### 4.2. Solid-State Fermentation (SSF)

The scale-up challenges thus far discussed are specifically referring to submerged liquid fermentation, be it conventional or precision. Solid-state fermentation (SSF), which has been in existence for centuries, is another option that is relevant in the context of operational cost, resources, and effectiveness. SSF-derived foods such as kimchi, tempeh, and koji are some of the familiar products. Indeed, SSF is amenable to customization and has low operational costs. Due to its significantly reduced moisture requirements (50–60% depending on applications), SSF has tremendous potential for enzyme production. While various small, pilot-scale, and large-scale SSF bioreactors are already available commercially, efforts have been made to develop pilot-scale SSF bioreactors specifically geared toward the production of enzymes. For example, a packed-bed SSF bioreactor was designed for the production of pectinases and lipases from fungi [107]. Requiring minimal water, SSF is well-suited for the use of the substrate itself as a matrix for immobilizing the microbial cells and producing enzymes that can alter the texture, flavor and aroma profiles without the need for purifying enzymes to achieve the desired results; essentially the traditional approach to SSF. Nonetheless, studies have shown that enzymes can be recovered from SSF, although scale-up production still needs to be demonstrated. Using grape pomace and wheat bran as substrate, a mutant strain of *Aspergillus niger* 3T5B8 was shown to produce hydrolytic enzymes, which were subsequently used as a cocktail for the release of bioactive compounds from grape pomace [108]. Similarly, α-amylase and protease were produced and extracted at a laboratory scale using *Rhizopus oryzae* and waste bread as substrate [109]. In another study, SSF was used at the laboratory scale and pilot scale (600 L) to produce α-amylase from *Aspergillus oryzae* and edible oil as substrate [110]. These studies not only demonstrate the potential of SSF for enzyme production, but also for the use of waste feedstocks or residues readily available. With the advent of precision fermentation and the need to move towards more sustainable practices, SSF serves as an ideal platform for further exploring its potential for bioengineered enzyme production and the use of inexpensive feedstocks commonly available. Recently, CRISPR/Cas9-edited genes in yeast were demonstrated to produce higher CO_2_ for bread leavening and reduced acrylamide in baked bread and potato chips [111]. Although this study was at a laboratory scale, it augurs well for the use of edited enzyme genes in microorganisms to catalyze specific changes in foods via SSF, besides further exploring a solid-state precision fermentation (SSPF) approach for the large-scale production of bioengineered enzymes.

SSF as an economically more sustainable approach for the production of enzymes is not without its limitations. One of its major limitations is the availability of large-scale systems. Also, at larger scales heat transfer and aeration can prevent the optimal growth of microorganisms. Due to the minimal use of moisture, extraction of secreted enzymes can be challenging. Therefore, methods need to be developed to process enzymes out of the solid substrates [112]. To reduce the duration of the SSF, high-density inoculum also needs to be used. Thus, the production of the latter introduces another additional step in the process, which may preclude any cost or time-saving. Therefore, fungal species for the production of enzymes by SSF appear to be more amenable than bacterial species. Furthermore, fungal species are more versatile and effective for the use of agricultural feedstocks as substrates. For example, using agricultural residues, cellulase production at high titres was demonstrated in SSF from *Aspergilllus tubingensis* NKBP-55 [113]. In fact, many agricultural feedstocks have been used as substrates with a variety of fungal species for the production of enzymes using SSF [112], and as mentioned above with investment of resources SSF can become a more effective approach to enzyme production in the precision fermentation landscape.

## 5. Concluding Remarks

Microbial food fermentation has been practiced for thousands of years. However, unbeknownst in those days was the fact that many of the textural, aromatic, and flavor profile changes were due to enzyme-catalyzed modifications. Subsequently, the realization that these enzymes could be produced, isolated, and purified at an industrial scale through microbial fermentation, led to the emergence of a completely new food processing approach. However, the efficiency of wild-type enzyme catalytic activity tended to be low. Thus, engineered enzymes for improved catalytic activity became more prevalent. Beyond the traditional rational design, directed evolution, and semi-rational design approaches, the advent of targeted gene editing, AI-based in silico design, and testing approaches further broadened the scope of manipulation of enzymes for improved activity, functionality, stability, and efficiency. However, the bottleneck currently lies in the scale-up production of these enzymes due to limited capacity, know-how, and accessibility. To derive maximum value from the novel enzymes emanating from synthetic biology and precision fermentation, it is imperative that scale-up challenges be addressed. Additionally, the cost of production could be reduced by exploring SSF approaches and inexpensive feedstocks.

## Figures and Tables

**Table 1:** Examples of engineered enzymes and their effects in food applications.

Engineered Enzyme	Source	Method	Effect	Example of Application	References
Directed evolution					
α-amylase Novamyl	*Bacillus* sp. (TS-25)	Error-prone PCR	Increased thermostability at acidic pH	Bakery	[8]
α-amylase	*Bacillus licheniformis*	Error-prone PCR	Increased thermostability	Bakery	[12]
α-amylase	*Rhizopus oryzae*	Multiple sequence alignment-based site-directed mutagenesis	Improved the thermostability and acid resistance	Starch industry and brewery	[9]
α-amylase	*Bacillus cereus* GL96	Combining computer-aided directed evolution and site-directed mutagenesis	Increased thermostability (70 °C) and stability over a range of pH from 4 to 11)	Bakery	[13]
Xylanase (reBaxA50)	*Bacillus amyloliquefaciens*	Error-prone touchdown PCR	Increased catalytic efficiency and stability under thermal and extreme pH	Biorefinery	[14]
Xylanase	*Bacillus amyloliquefaciens xylanase A (BaxA) and Thermomonospora fusca*	DNA shuffling	Increased specificity and catalytic efficiency	Production of prebiotic xylo-oligosaccharides	[15]
Lipase	Penicillium *cyclopium*	Error-prone PCR	Enhanced thermostability	Bakery and dairy	[16]
Lipase	*Pseudomonas fluorescens*	Error-prone PCR	Enhanced alkali stability	Bakery and dairy	[17]
β-galactosidase	*Escherichia coli*	Error-prone PCR	Increased activity	Milk processing	[18]
Alkaline protease	*Bacillus alcalophilus*	Error-prone PCR	Increased cold adaptation	Cold-temperature food processing	[19]
Transglutaminase	*Streptomyces mobaraensis*	Directed Evolution and Molecular Dynamics Simulation	Improved thermostability and specific activity	Bakery	[20]
Rational design					
Serine peptidase	*Pseudomonas aeruginosa*	Site-directed mutagenesis	Improved thermal stability and catalytic efficiency	Dairy	[21]
Xylanase	*Streptomyces*	Site-directed mutagenesis	Enhanced substrate specificity	Bread making	[22]
β-glucanase	*Bacillus terquilensis*	Site-directed mutagenesis	Enhanced thermostability	Cereal-based sector	[23]
β-glucanase	*Bacillus* sp. SJ-10	Site-directed mutagenesis	Enhanced catalytic efficiency, halostability, and thermostability	Hemicelluloses hydrolysis	[24]
Lipase isozymes	*Candida rugosa*	Site-directed mutagenesis	Increased catalytic efficiency	Food emulsifiers	[25]
Cel9A-68 cellulase	*Thermobifida fusca*	Computer-aided enzyme simulation	Increased catalytic activity	Brewery and wine	[26]
Lipase	*P. aeruginosa PAO1*	Computational “reverse engineering”	Increased activity and stability	Dairy products such as cheese	[27]
GH11 xylanase	*Neocallimastix patriciarum*	Site-directed mutagenesis guided by sequence and structural analysis	Improved thermostability and kinetic efficiency	Cereal processing	[28]
GH11 xylanase	*Bacillus* sp. *strain (T82A)*	Site-saturation mutagenesis	Increased catalytic activity	Cereal processing	[29]
GH11 xylanase	*Aspergillus niger*	Virtual mutation and molecular dynamics simulations	Increased catalytic activity and thermostability	Cereal processing	[30]
Semi-rational design					
Type II ASNase	*Bacillus licheniformis*	Structural alignment and molecular dynamic simulation	Increased catalytic efficiency, structure stability, and substrate binding	Fried potato products, bakery products, and coffee	[31]
Sucrose phosphorylase	*Bacillus licheniformis*	Semi-rational mutagenesis and low-throughput	Increased selectivity	Confectionery products	[32]
Cellobiose 2-epimerase	*Caldicellulosiruptor saccharolyticus*	Computational prediction performance and molecular dynamics simulation	Improved thermostability and catalytic efficiency	Production of lactose-based prebiotics	[33]

## Data Availability

Not applicable.
